# Expanding the Known Functional Repertoire of the Human Cytomegalovirus pp71 Protein

**DOI:** 10.3389/fcimb.2020.00095

**Published:** 2020-03-12

**Authors:** Robert F. Kalejta, Emily R. Albright

**Affiliations:** McArdle Laboratory for Cancer Research, Institute for Molecular Virology, University of Wisconsin – Madison, Madison, WI, United States

**Keywords:** cytomegalovirus, HCMV, pp71, tegument, chromatin, latency

## Abstract

The human cytomegalovirus pp71 protein is packaged within the tegument of infectious virions and performs multiple functions in host cells to prime them for productive, lytic replication. Here we review the known and hypothesized functions of pp71 in regulating proteolysis, infection outcome (lytic or latent), histone deposition, transcription, translation, immune evasion, cell cycle progression, and pathogenesis. We also highlight recent advances in CMV-based vaccine candidates informed by an improved understanding of pp71 function.

## Introduction

Herpesviruses such as human cytomegalovirus (HCMV) have two distinct infection types, productive (lytic) and latent. A key step in determining which infection type will be initiated upon infection is the transcription of the viral immediate early (IE) genes, which must be activated to initiate productive infection but suppressed when latency is established. The promoter that drives the transcription of the major IE genes, the Major Immediate Early Promoter (MIEP), is not fully constitutively active in the context of the viral genome, but rather is transactivated by a virally encoded protein that is packaged within the tegument layer of infectious virions and is co-delivered to cells upon virion entry. For HCMV, that tegument transactivator is the pp71 protein that stimulates the MIEP in reporter assays (Liu and Stinski, 1992) and translocates to the nucleus in lytically infected cells (Hensel et al., 1996) to activate the MIEP and induce the transcription of the genes encoding the viral IE1 and IE2 proteins. A pp71-null HCMV recombinant (Bresnahan and Shenk, 2000) has a major defect in IE gene expression and productive replication. During a productive, lytic infection, tegument-delivered pp71 initiates and sustains MIEP transcription long enough for the *de novo* synthesized IE1 protein to promote prolonged lytic phase gene expression (Vardi et al., 2018) and the completion of productive replication.

**Figure 1:**
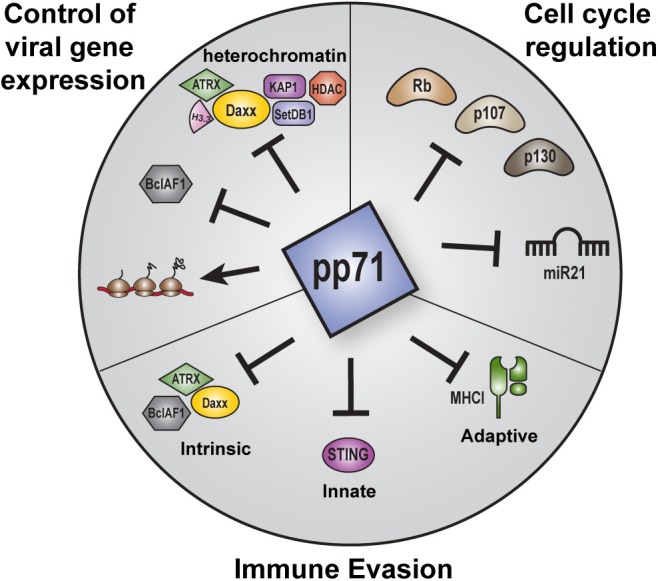
Overview of the major roles of pp71 during HCMV infection. Major functions of pp71 include control of viral gene expression, immune evasion, and cell cycle regulation. pp71 regulates viral gene expression by preventing the assembly of transcriptionally repressive heterochromatin on viral genomes, inhibiting intrinsic cellular factors, and promoting translation. pp71 contributes to immune evasion by inhibiting components of the intrinsic, innate, and adaptive immune responses. pp71 also controls cell cycle progression by inhibiting the Rb family of tumor suppressor proteins and the onco-microRNA miR21.

But pp71 is not simply an activator of IE transcription at the start of lytic infections. This protein also controls viral chromatin states, evasion of intrinsic, innate, and adaptive immunity, latency establishment, the cell cycle, and likely other viral and cellular processes that contribute to HCMV infections (Figure 1). Here are discussed the numerous activities of pp71, with particular attention to features newly-discovered or not sufficiently addressed in previous reviews (Kalejta, 2004, 2008a,b; Saffert and Kalejta, 2008; Penkert and Kalejta, 2011, 2012).

## pp71 Modifications, Interactions, Functions, and Activities

pp71, encoded by the UL82 gene, is a phosphorylated protein with an apparent molecular weight of 71 kDa. It is phosphorylated on multiple residues (Shen et al., 2008), and these residues, when phosphorylated or not, control the sub-cellular localization of ectopically expressed pp71. It is unclear how phosphorylation affects the localization or function of pp71 when naturally expressed from the viral genome during infection. pp71 is also SUMOylated (Hwang and Kalejta, 2009), although a role for this modification has not been demonstrated. pp71 was also recently reported to undergo protein S-nitrosylation, which may modify its ability to impair host innate immune defenses (see below) (Nukui et al., 2020). Other post-translational modifications of pp71 have not been reported.

Proteins that interact with pp71 have been determined by multiple methods, including yeast two-hybrid screening, pulldown-mass spectrometry, and co-immunoprecipitation. Functions for most of the viral (Phillips and Bresnahan, 2011; Nobre et al., 2019) and cellular (Hofmann et al., 2002; Lee et al., 2012; Nobre et al., 2019) interactions are not known. The major interactors of pp71, for which functions have been established and are described below, include Daxx, STING, and the retinoblastoma (Rb) family of tumor suppressors.

The major functions of pp71 appear to be the control of viral IE transcription, regulation of the cell cycle, and immune evasion. pp71 has no known enzymatic activity, although the protein shares homology with dUTPase family members while lacking residues critical for catalytic function (Davison and Stow, 2005). The enzymatic function most associated with pp71 is proteolysis, where pp71 mediates the proteasome-dependent degradation of at least a subset of the proteins to which it binds, including Daxx (Saffert and Kalejta, 2006), BclAF1 (Lee et al., 2012), and the Rb family members (Kalejta et al., 2003). While pp71-mediated degradation requires the ubiquitin-binding 19S regulatory particle of the proteasome (Winkler et al., 2013), its substrates are degraded in a ubiquitin-independent manner (Kalejta and Shenk, 2003a; Hwang and Kalejta, 2007). The extent to which other pp71 interactors are degraded and the mechanism of the ubiquitin-independent degradation remain to be revealed.

In addition to mediating the proteolysis of other proteins, pp71 itself is subject to proteolysis by Granzyme M (Van Domselaar et al., 2010). Granzymes are secreted by immune cells. Their entry into infected cells is supported by co-secreted perforin. Within infected cells, granzymes cleave protein substrates and eventually lead to cell death by apoptosis. Cleavage of pp71 by granzyme M inhibits its ability to activate an MIEP reporter construct (Van Domselaar et al., 2010), and thus would presumably inhibit HCMV productive replication. Granzyme M cleaves pp71 after its leucine amino acid at position 439 (Van Domselaar et al., 2010) in a region of pp71 lacking known functional significance. A virus in which the leucine at position 439 of pp71 is substituted with alanine (HCMV-pp71-L439A) to render it non-cleavable by Granzyme M grows without complementation in fibroblasts *in vitro* (Laura Winkler and Rob Kalejta, unpublished observations), leading to questions of why this residue that confers immune susceptibility is not selected against. Perhaps granzyme M-mediated cleavage inactivates pp71 in the context of viral latency during which pp71 function is detrimental to the virus (see below). HCMV may use pp71 proteolysis as a means to determine if the local immune environment is inhospitable to productive replication and, if so (if pp71 is cleaved by granzyme M), to eschew reactivation. Therefore, perhaps a leucine at position 439 of pp71 has actually been selected for, rather than being selected against.

## Entry and Uncoating

Although it plays no known or expected role in the process of HCMV entry, the fate of the pp71 protein delivered to cells by infectious virions plays a major role in determining the outcome of an HCMV infection. In differentiated cells such as fibroblasts and epithelial cells, tegument-delivered pp71 quickly migrates to and remains in the nucleus (Hensel et al., 1996; Weng et al., 2018). However, in the undifferentiated cell line models used to study latency (Lee et al., 2019), as well as the primary CD34+ hematopoietic progenitor cells that support both *in vitro* and *in vivo* latency, tegument-delivered pp71 remains trapped in the endosomes through which the virus enters, never reaching the nucleus (Lee and Kalejta, 2019). These different sub-cellular localizations of tegument-delivered pp71 have profound impacts on the ability of the protein to affect viral transcription (see the “Chromatin and Transcription” and “Latency” sections below).

## Chromatinization and Transcription

pp71 plays a key role in promoting the transcription of viral IE genes at the onset of productive replication. Recombinant viruses lacking pp71 have impaired IE gene expression and a pronounced replication defect, particularly at low multiplicities of infection (Bresnahan and Shenk, 2000; Cantrell and Bresnahan, 2005). Although pp71 had been shown to transactivate the MIEP (Liu and Stinski, 1992), the mechanism it employed to do so remained elusive for over a decade. A breakthrough into how pp71 transactivates IE transcription came when pp71 was found to interact with the cellular protein Daxx (Hofmann et al., 2002). Daxx, along with PML and Sp100, is a prominent component of PML Nuclear Bodies (PML-NBs) originally observed as nuclear dots reacting with auto-antigen (Rothfield and Rodnan, 1968; Bernstein et al., 1984; Fritzler et al., 1984) and Sp100 antibodies (Szostecki et al., 1990). At the time pp71 was found to interact with Daxx, PML-NBs were a conundrum because, while the structures were eventually disrupted during productive infections with herpesviruses (Maul et al., 1993; Kelly et al., 1995) and adenoviruses (Korioth et al., 1995), viral genomes seemed to specifically localize to these structures (Ishov and Maul, 1996) and to initiate viral transcription adjacent to them (Ishov et al., 1997). Thus, PML-NBs had features that could be seen as pro-viral, and others that could be seen as anti-viral (Maul, 1998). Importantly, pp71 mutants that failed to interact with Daxx also failed to activate an MIEP reporter (Hofmann et al., 2002), showing that the interaction between pp71 and Daxx was critical for pp71's transactivation function. An increase in MIEP reporter activity and pp71 localization to PML-NBs was observed when Daxx and pp71 were co-transfected, leading to a model in which Daxx played a pro-viral role by recruiting pp71 to PML-NBs where pp71 transactivated viral gene expression (Hofmann et al., 2002). The co-localization of pp71 and Daxx at PML-NBs was subsequently confirmed (Ishov et al., 2002; Marshall et al., 2002), leading to the speculation that pp71 facilitated HCMV genome deposition and subsequent transcription at PML-NBs.

Although early studies generated models in which PML-NBs were acting in a pro-viral manner to support HCMV infection (Hofmann et al., 2002; Ishov et al., 2002; Marshall et al., 2002), there were also data indicating that PML-NB proteins could possibly be antiviral. The inhibition of cellular histone deacetylases (HDACs), some of which partially localize to PML-NBs, improved herpesvirus lytic phase gene expression (Murphy et al., 2002; Tang and Maul, 2003). Furthermore, Daxx was found to recruit HDACs to integrated copies of Avian Sarcoma Virus (ASV) -based reporters, and to repress their transcription (Greger et al., 2005). In addition, PML-NB components were observed to re-localize to incoming Herpes Simplex Virus Type 1 (HSV-1) genomes (Everett and Murray, 2005) as opposed to viral genomes searching for existing PML-NBs. Indeed, later work (Diner et al., 2016; Everett, 2016) confirmed that PML-NB proteins, as well as the DNA sensor IFI16, are mobilized to incoming viral genomes during infections with HSV-1 and HCMV. Thus, early studies also implied an inhibitory role of PML-NB proteins against viral replication.

**Figure 2:**
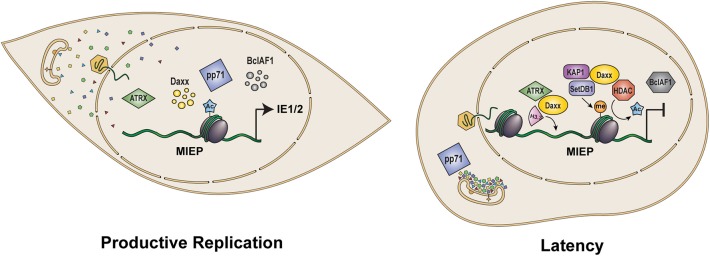
Role of pp71 in control of viral gene expression and latency. Upon infection of terminally differentiated cells such as fibroblasts and epithelial cells (Left), tegument delivered pp71 localizes to the nucleus where it inactivates intrinsic defense proteins Daxx, ATRX, and BclAF1, limiting histone deposition and heterochromatin formation on the viral genome to allow for the transcriptional activation of viral IE genes and productive viral infection. In contrast, following infection of incompletely differentiated myeloid cells such as CD34+ HPCs and monocytes (Right), tegument-delivered pp71 remains trapped in cytoplasmic endosomes. As a result, Daxx is not degraded and facilitates the histone deposition and assembly of repressive heterochromatin on viral genomes in conjunction with ATRX and the KAP1/SetDB1/HDAC co-repressor complex. Heterochromatin assembly results in repression of the MIEP allowing for the establishment of latency. MIEP: Major Immediate Early Promoter. Ac: histone acetylation. Me: histone methylation.

In addition to binding Daxx, independent work showed that pp71 also bound to other cellular transcriptional repressors, namely the Rb family of tumor suppressors, Rb, p107, and p130, and induced their proteasome-dependent, ubiquitin-independent degradation (Kalejta and Shenk, 2003a,b; Kalejta et al., 2003). When the effect of pp71 on Daxx steady state levels was examined, it became clear that pp71 was sufficient to degrade Daxx when ectopically expressed in cells, and necessary for Daxx degradation during HCMV infection (Saffert and Kalejta, 2006). Furthermore, Daxx knockdown promoted HCMV IE gene expression (Cantrell and Bresnahan, 2006; Preston and Nicholl, 2006; Saffert and Kalejta, 2006; Woodhall et al., 2006). These experiments were some of the first to use RNA interference to study HCMV infection (Wiebusch et al., 2004; Wills et al., 2005) and provided the first definitive proof that a PML-NB protein (Daxx) was not pro-viral as had been proposed (Hofmann et al., 2002; Ishov et al., 2002), but was actually anti-viral. Daxx was described as an inhibitor of intrinsic immunity against HCMV (Saffert and Kalejta, 2006). These negative effects of Daxx on HCMV infections were quickly confirmed and extended to the additional PML-NB proteins (Figure 2) ATRX, BclAF1, PML, and Sp100 by multiple independent groups (Cantrell and Bresnahan, 2006; Everett et al., 2006; Preston and Nicholl, 2006; Tavalai et al., 2006; Lukashchuk et al., 2008; Adler et al., 2011; Lee et al., 2012). Since then, the inhibitory effect of PML-NBs on viral infections has been codified (Everett and Chelbi-Alix, 2007; Scherer and Stamminger, 2016), and we now appreciate that all human herpesviruses likely encode a tegument protein that can inactivate intrinsic defenses (Lieberman, 2016).

Together, Daxx and ATRX form a histone chaperone complex that deposits the replication-independent histone variant H3.3 onto DNA (Drané et al., 2010; Lewis et al., 2010). Although devoid of histones within virions, HCMV genomes are rapidly assembled into nucleosomes upon entry into the nucleus of host cells during both productive and latent infection (Murphy et al., 2002; Nitzsche et al., 2008). This chromatinization process requires neither viral gene expression, nor viral DNA replication (Nitzsche et al., 2008; Albright and Kalejta, 2016). As this initial chromatinization of infecting viral genomes is mediated by intrinsic defense proteins, it may represent a prescribed cellular response aimed at repressing expression of foreign or non-nucleosomal DNA (Penkert and Kalejta, 2012; Knipe, 2015). Histone H3.3 is associated with HCMV genomes during both productive and latent infection (Albright and Kalejta, 2016). Knockdown of Daxx prior to latent infection of THP-1 monocytes decreased H3.3 occupancy of viral genomes and increased the steady state levels of viral IE1/2 mRNAs that are normally kept low or absent during latency (Albright and Kalejta, 2016). Interestingly, the replication-dependent histones H3.1 and H3.2 are also deposited onto latent viral genomes in a Daxx-dependent manner (Albright and Kalejta, 2016). H3.1 and H3.2 are also deposited on viral genomes during productive infection of fibroblasts (Albright and Kalejta, 2016). Consistent with a role for pp71 in controlling histone deposition on viral genomes, a recombinant pp71-null virus has increased histone H3 occupancy during infection of fibroblasts (Albright and Kalejta, unpublished observations).

Beyond its role as a histone chaperone, Daxx also interacts with histone deacetylases (HDACs) as well as with the co-repressor KAP1 and H3K9 histone methyltransferase SetDB1 to promote heterochromatin formation and transcriptional repression (Elsässer et al., 2015; Hoelper et al., 2017). During both productive and latent HCMV infection, histones associated with viral genomes initially bear marks of transcriptionally repressive heterochromatin, including hypoacetylated histones and methylation at H3K9 and H3K27 (Murphy et al., 2002; Groves et al., 2009; Sourvinos et al., 2014; Lee et al., 2015). Knockdown of Daxx resulted in increased levels of acetylated histones and decreased levels of H3K9methylation marks associated with HCMV genomes during both lytic (Woodhall et al., 2006) and latent (Albright and Kalejta, unpublished observations) HCMV infection. Both KAP1 and SetDB1 are associated with latent viral genomes and knockdown of KAP1 resulted in decreased H3K9me3, and increased IE1/2 transcript levels and productive replication following infection of CD34+ HPCs in which HCMV normally establishes a latent infection (Rauwel et al., 2015). Thus, inhibition of histone deposition and heterochromatin formation by pp71-mediated inactivation of Daxx and ATRX seems to promote a viral chromatin structure conducive to transcription and is one way in which pp71 stimulates productive phase gene expression.

In addition to mediating the degradation of Daxx, pp71 also induces Daxx SUMOylation (Hwang and Kalejta, 2009). The significance of Daxx SUMOylation to HCMV infection is unclear as this modification of Daxx is not required for Daxx degradation by pp71 nor does it appear to have a substantial impact on induction of IE gene expression, at least in fibroblasts (Hwang and Kalejta, 2009). However, Daxx SUMOylation prevents it from binding to NFkB and from inhibiting NFkB acetylation and activation (Croxton et al., 2006; Park et al., 2007; Kim et al., 2017). The HCMV MIEP contains multiple binding sites for NFkB (Meier and Stinski, 1996) and NFkB is a well-established activator of MIEP activity (DeMeritt et al., 2004; Liu et al., 2010; Yuan et al., 2015; Hancock and Nelson, 2017), although the binding sites for NFkB in the MIEP are not required for productive replication in fibroblasts (Gustems et al., 2006). Thus, pp71-mediated Daxx SUMOylation may contribute to NFkB-mediated activation of the MIEP which could be important for efficient IE gene expression in certain contexts.

## Latency

The Daxx-mediated silencing of the MIEP that inhibits productive, lytic infection (see above) actually supports the establishment of latency (Figure 2). During latency, productive replication is inhibited, in part, by keeping the transcription and translation of the viral IE1 and IE2 genes low or absent (Sinclair and Reeves, 2013). Daxx knockdown activates productive phase gene expression during latency in undifferentiated myeloid cells (Saffert and Kalejta, 2007; Saffert et al., 2010), indicating that Daxx silences IE gene expression to support HCMV latency. Of note, transient knockdown of Daxx was not sufficient to promote IE gene expression from the Toledo strain of HCMV in the NT2 model of latency (Groves and Sinclair, 2007), likely because low passage strains retain additional viral factors capable of suppressing the MIEP independent of Daxx and HDAC activity that may mask the effects of Daxx knockdown (Saffert et al., 2010; Lee et al., 2015). Therefore, despite being anti-viral against productive infection, Daxx is actually pro-viral for latency establishment (Saffert and Kalejta, 2008). However, this pro-viral role for Daxx (and perhaps other PML-NB proteins) during latency must be overcome to permit reactivation to productive replication. Interestingly, a viral protein encoded by the DNA strand complementary to, and partially overlapping with the UL82 gene that encodes pp71, seems to be responsible for inactivating PML-NBs during reactivation. The LUNA protein (Latency Unique Natural Antigen) (Bego et al., 2011) is expressed from a transcript antisense to the viral UL81 and UL82 genes (Bego et al., 2005) that, despite the name, is not unique to latency, but is expressed during lytic infection as well (Keyes et al., 2012). LUNA is required for reactivation (Keyes et al., 2012) and possesses a deSUMOylase activity that disrupts PML-NBs during reactivation (Poole et al., 2018). The revelation that PML-NBs are disrupted during reactivation supports previous results showing that a major PML-NB component, Daxx, silences viral productive phase gene expression during latency (Saffert and Kalejta, 2007, 2008; Saffert et al., 2010).

Daxx is able to silence productive phase HCMV transcription during latency because it is spared from degradation by tegument-delivered pp71, which remains in the cytoplasm of the undifferentiated cells that support HCMV latency (Saffert and Kalejta, 2007; Saffert et al., 2010; Albright and Kalejta, 2013; Penkert and Kalejta, 2013; Lee and Kalejta, 2019; Lee et al., 2019). The cytoplasmic localization of tegument-delivered pp71 has been demonstrated in all cell types that support experimental HCMV latency *in vitro* (Lee et al., 2019), including primary CD34+ hematopoietic progenitor cells (Saffert et al., 2010; Lee and Kalejta, 2019). The nuclear import of tegument-delivered pp71 is blocked (Penkert and Kalejta, 2010) and the protein remains associated with the endosomes through which HCMV virions enter the undifferentiated myeloid cells in which latency is established (Lee and Kalejta, 2019; Lee et al., 2019). While genome-containing capsids are liberated from the endosomes through which the virus enters in both differentiated cells (productive infection) and undifferentiated cells (latency) (Lee and Kalejta, 2019; Lee et al., 2019), it appears that an as yet unidentified factor expressed only in differentiated cells (Penkert and Kalejta, 2010) is required for pp71 to escape endosomes, migrate to the nucleus, transactivate the MIEP, and initiate productive infection. The importance of pp71-mediated Daxx degradation and the subsequent initiation of viral transcription for successful productive replication, and preventing these events for the establishment of latency, makes understanding the mechanism of pp71 cytoplasmic sequestration paramount.

While it is clear that pp71 plays a crucial role in initiating viral IE gene expression at the onset of a *de novo* lytic infection, whether it plays a similar role during reactivation from latency remains to be determined. As discussed above, tegument-delivered pp71 localizes to endosomes within the incompletely differentiated cells in which HCMV establishes and maintains latency (Lee and Kalejta, 2019; Lee et al., 2019). Presumably, HCMV can maintain latency for weeks to months to years prior to reactivation. It is improbable that tegument-delivered pp71 would remain present and capable of inducing reactivation after such an extended period of time. Interestingly, studies of the reactivation of HSV1 have suggested a bi-phasic process of reactivation where a transient, low-level de-repression of the entire viral genome is followed by the classical temporally regulated cascade of lytic gene expression leading to viral genome amplification and productive replication (Du et al., 2011; Kim et al., 2012; Cliffe et al., 2015). If such a process also occurs during HCMV reactivation, it could allow for the *de novo* expression of pp71 that makes the protein capable of translocating to the nucleus (Saffert and Kalejta, 2007; Saffert et al., 2010), inducing Daxx degradation, and trans activating the MIEP to facilitate sustained lytic-phase gene expression and full-fledged reactivation. Such a scenario would be analogous to that of the HSV-1 tegument transactivator VP16 that is expressed *de novo* prior to reactivation and localizes to the nucleus to promote efficient reactivation (Thompson et al., 2009; Kim et al., 2012). However, it is also possible that activation of IE gene expression during HCMV reactivation occurs through mechanisms that circumvent the requirement of pp71, either because LUNA-mediated disruption of PML-NBs (Poole et al., 2018) is sufficient to depress the MIEP and/or through the use of alternative promoter sequences to drive transcription of IE1/2 during reactivation (Collins-McMillen et al., 2019) whose dependency on pp71 is unknown. Determining what role, if any, pp71 plays during reactivation warrants additional investigation.

## Translation

pp71 was recently demonstrated to bind RNA (Lenarcic et al., 2015), a finding foreshadowed by the ontology of its interacting proteins (Lee et al., 2012). In fact, one member of the pp71 interactome, the RNA helicase DHX9, was found to have enhanced association with RNA in HCMV infected cells (Lenarcic et al., 2015), although the necessity of pp71 for this increased interaction was not tested. pp71 was found in polysomes and enhanced overall protein translation in transiently transfected HeLa cells (Lenarcic et al., 2015). Co-expression of UL35a, an HCMV protein that interacts with pp71 (Schierling et al., 2004; Salsman et al., 2011), further increased protein synthesis. Interestingly, co-expression of pp71 induced a slower migrating form of UL35a that may be consistent with SUMOylation (Lenarcic et al., 2015), indicating that, like its other binding partner Daxx (Hwang and Kalejta, 2009, 2011), pp71 may induce the SUMOylation of UL35a. The function of pp71 binding to DHX9 or RNA during viral infection remains to be examined.

## Immune Evasion

**Figure 3:**
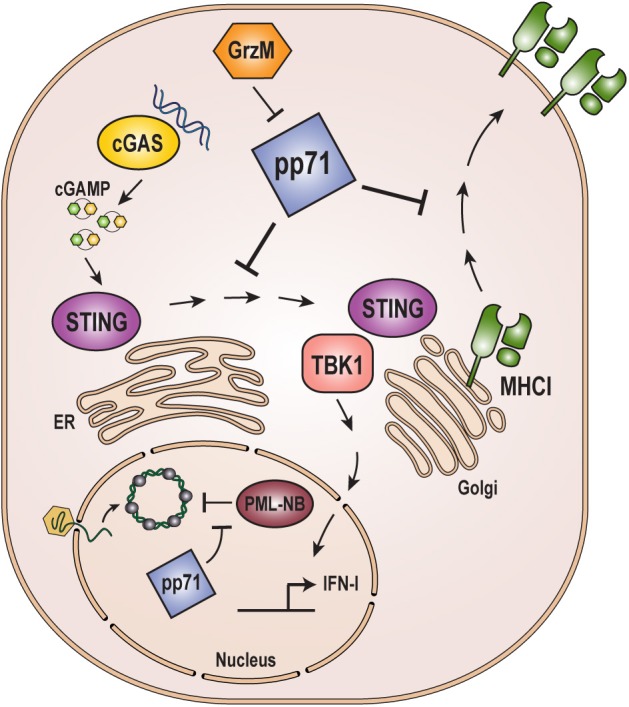
Role of pp71 in evading host immune responses. pp71 counteracts intrinsic, innate, and adaptive immune responses. Incoming viral genomes are targeted by the intrinsic immune defense mediated by PML-NB proteins resulting in heterochromatinization and transcriptional silencing. pp71 contributes to evasion of intrinsic defenses by inactivating PML-NB components Daxx, ATRX, and BclAF1 (see Figure 2). The cGAS-STING-TBK1 innate immune sensing pathway is triggered by cGAS recognition of dsDNA, such as viral genomes, which signals through STING and TBK1 to ultimately result in the upregulation of interferon-stimulated genes and inflammatory cytokines. pp71 inhibits this pathway by blocking the translocation of STING from the ER to the Golgi where it interacts with TBK1. Adaptive immune responses include presentation of viral antigens on the cell surface via MHC class I molecules. pp71 inhibits this presentation by impairing trafficking of MHC class I. pp71 is itself targeted by the immune system via cleavage by Granzyme M (GrzM). cGAMP: cyclic GMP-AMP.

Human immune systems are comprised of intrinsic, innate, and adaptive branches, and pp71 regulates all three (Figure 3). Intrinsic immunity (Bieniasz, 2004) is mediated by constitutively-expressed proteins that act in a direct anti-viral manner. For herpesviruses like HCMV, a major intrinsic immune defense measure is the PML-NB protein-mediated chromatinization and transcriptional silencing of viral genomes (Tavalai and Stamminger, 2009). Indeed, Daxx was the first intrinsic immune defense identified to target a herpesvirus (Saffert and Kalejta, 2006), and is neutralized by pp71-mediated proteasome-dependent, ubiquitin-independent degradation (Hwang and Kalejta, 2007) as described above (see “Chromatin and Transcription”).

Innate immunity represents a broad set of responses activated after viral infections are detected through their pathogen-associated molecular patterns (PAMPs) by pattern recognition receptors (PRRs) (Chan and Gack, 2016; Ni et al., 2018). A major innate immune defense activated upon DNA virus infection is mediated by the cGAS-STING-TBK1 pathway (Ahn and Barber, 2019). Cyclic GMP-AMP synthase (cGAS) synthesizes cyclic GMP-AMP (cGAMP) when bound to dsDNA, such as infecting viral genomes. cGAMP in turn binds and activates the Stimulator of Interferon Genes (STING) triggering the phosphorylation and activation of TANK-binding kinase-1 (TBK1). TBK1 then activates cellular transcription factors to promote the expression of a series of antiviral, interferon-stimulated genes and inflammatory cytokines. pp71 disrupts this pathway by binding to STING and preventing its required sub-cellular translocation from the ER to the Golgi and its interaction with TBK1 (Fu et al., 2017). The pp71-mediated inactivation of STING has a quantitatively smaller positive effect on HCMV replication than does pp71-mediated inactivation of Daxx (Cantrell and Bresnahan, 2006), likely because there are multiple HCMV proteins that inactivate STING and other innate immune pathways (Stempel et al., 2019), but pp71 is the only known HCMV protein to counteract the inhibitory effects of Daxx. It was recently reported that pp71 is modified by S-nitrosylation at Cysteine 218 within its Rb-binding LxCxD motif (Nukui et al., 2020). Modification at this residue appears to impair the ability of pp71 to target the cGAS-STING-TBK1 pathway as a pp71 mutant that cannot be S-nitrosylated at this site more potently suppresses STING-mediated innate immune responses in the context of both ectopic expression and productive viral infection (Nukui et al., 2020). As discussed above (see “pp71 Modifications, Interactions, Functions, and Activities”), pp71 can be cleaved by Granzyme M (Van Domselaar et al., 2010), a protease secreted by cells of the innate and adaptive immune systems. Emerging evidence indicates that Granzyme M-mediated cleavage of pp71 also partially restores the cGAS-STING-TBK1 pathway in HCMV infected cells (Niels Bovenschen, personal communication), revealing an additional means through which innate and adaptive immunity might impede productive HCMV replication.

Adaptive immunity is a highly-specific response to unique antigens encoded by pathogens that not only acts in a direct, antiviral way to clear virus particles and kill infected cells, but also creates immunologic memory that can respond faster and stronger upon a second infection with the same pathogen. HCMV has multiple methods to avoid or neutralize adaptive immune responses (Noriega et al., 2012). One such method is through the pp71-mediated downregulation of the cell surface expression of Major Histocompatibility Complex (MHC) class I molecules (Trgovcich et al., 2006). MHC class I molecules present internal peptides on the cell surface. If engaged by a cytotoxic T cell of the adaptive immune system that recognizes the presented epitope, the cell is killed, halting the viral infection. pp71 may prevent MHC class I trafficking to or through the Golgi Apparatus, thus preventing its cell surface expression (Trgovcich et al., 2006). It is unclear whether or not a similar mechanism may be used to alter the trafficking of the innate immunity component STING and the adaptive immunity component MHC class I. Interestingly, tegument-delivered pp71 increases MHC class I presentation of peptides derived from the HCMV immediate early protein IE1 (Hesse et al., 2013), whose gene is transactivated by pp71. Perhaps the pp71-mediated, immune suppressive downregulation of MHC class I cell surface expression diminishes the immuno-stimulatory effect of pp71-mediated activation of viral transcription.

## Cell Cycle

**Figure 4:**
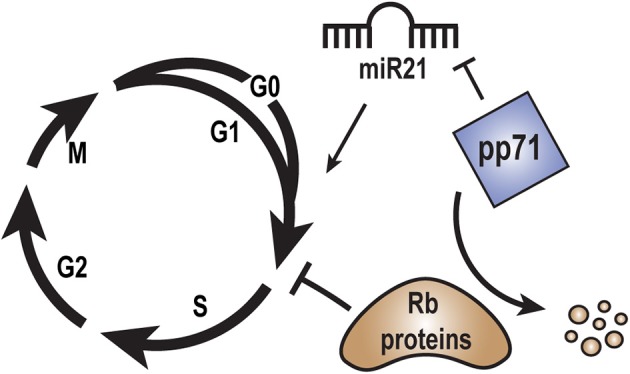
Role of pp71 in regulation of the cell cycle. Rb family members block cell cycle progression from G1 into S-phase. pp71 promotes cell cycle progression from G1 into S-phase by binding to and inducing the proteasome-dependent, ubiquitin-independent degradation of the Rb proteins. MicroRNA miR21 may promote cell cycle progression by down regulating tumor suppressor proteins. pp71 expression may inhibit miR21 levels in order to fine tune cell cycle control.

pp71 drives quiescent, G0 cells into the S phase (Kalejta et al., 2003) by inducing the proteasome-dependent, ubiquitin-independent degradation of the Rb family of tumor suppressors (Kalejta and Shenk, 2003a), Rb, p107, and p130 (Figure 4). An Rb-binding LxCxD motif within pp71 is required for this cell cycle induction. pp71 also accelerates cells through the G1 phase of the cell cycle (Kalejta and Shenk, 2003b) through a mechanism largely independent of its LxCxD motif. The ability of pp71 to stimulate cell cycle progression in multiple ways may be important for efficient viral productive replication, and may contribute to proliferative diseases associated with HCMV infection, including cardiovascular diseases and cancer.

The Rb protein is degraded by pp71 at the start of a productive HCMV infection (Hume et al., 2008), but reaccumulates at later times as a series of slower migrating forms due to phosphorylation by the viral cyclin dependent kinase like (v-Cdk) protein UL97 (Hume et al., 2008; Prichard et al., 2008). The significance of the reappearance of Rb was unknown until it was demonstrated that HCMV replicates less efficiently in Rb knockdown cells (VanDeusen and Kalejta, 2015a,b). Another pp71 substrate, Daxx, is also degraded at early times but reappears later in infection (Saffert and Kalejta, 2006). Unlike Rb, however, Daxx knockdown slightly enhances productive replication of wild-type HCMV (Cantrell and Bresnahan, 2006). It is currently unclear if the ability of pp71 to degrade substrates late during infection is globally impaired, or if certain pp71-substrates simply re-accumulate (e.g., pp71 fails to bind phosphorylated Rb species, so UL97-mediated phosphorylation may protect Rb from pp71-mediated degradation).

miR21 is a micro RNA highly associated with human cancers (Wu et al., 2015). Most miR21 targets are tumor suppressor proteins, and thus it is considered an onco-miR, although it also downregulates the cell cycle stimulatory CDC25A phosphatase (Wang et al., 2009). miR21 levels are decreased by 20% in U-251MG glioblastoma cells engineered to ectopically express pp71 (Fu et al., 2015). The role of miR21 or its downregulation by pp71 during HCMV infection remains unknown.

## Pathogenesis

HCMV genomes and proteins can be detected in glioblastoma multiforme (GBM) tumors (Cobbs et al., 2002; Dziurzynski et al., 2012; Ranganathan et al., 2012; Liu et al., 2017), and chemical or immunological modalities directed at HCMV are being explored as adjunct therapy for GBMs (Foster et al., 2017). Although pp71, like the oncoproteins of other DNA tumor viruses (Kalejta, 2004) inactivates the Rb tumor suppressors (Kalejta and Shenk, 2003a,b; Kalejta et al., 2003), there is no evidence that HCMV in general, or pp71 in particular, drives oncogenic cell division in virus-positive GBMs. However, in GBM cells, pp71 expression activates the NFkB pathway leading to increased expression of genes involved in the inflammatory response, tissue remodeling, and angiogenesis (Matlaf et al., 2013). Such a response could theoretically promote tumor cell survival and metastasis. Interestingly, mutations in Daxx, ATRX, or the histone H3.3 that they deposit are highly prevalent in pediatric glioblastomas (Schwartzentruber et al., 2012). As HCMV seropositivity increases with age (Bate et al., 2010), perhaps pp71-mediated inactivation of Daxx precludes the need for mutation of this pathway in adults with HCMV-positive tumors. Recently, Daxx and the Phosphatase and Tensin homolog (PTEN) tumor suppressor were shown to have a synthetic lethal relationship in GBMs, where Daxx knockdown impaired tumor growth in PTEN-negative but not PTEN-positive tumor cells (Benitez et al., 2017). Thus, it would be interesting to determine whether there is an inverse relationship between HCMV-positivity and PTEN mutation in adult GBMs.

As many as 1% of newborns are infected with congenitally-acquired HCMV infections that can cause life-long neurological problems including microcephaly, mental defects, and hearing loss (Britt, 2018; Permar et al., 2018). The virus can infect neural progenitor cells where pp71 expression results in slight decreases in the steady state levels of JAG1 and NICD1, components of the Notch pathway (Li et al., 2015). It is unclear if the modest downregulation observed affected the function of the Notch pathway for proliferation or differentiation in these cells, or if this downregulation by pp71 leads to any sequelae during congenital infections.

Finally, in addition to causing pathogenesis, vaccine approaches informed by research on the functions of pp71 are promising new weapons to treat or prevent HCMV-induced pathogenesis. Rhesus cytomegalovirus (RhCMV)-based vectors are being developed as vaccine candidates (Hansen et al., 2011) for multiple infections, including the Human Immunodeficiency Virus (HIV) that causes AIDS. RhCMV vectors expressing Simian Immunodeficiency Virus (SIV) antigens controlled and even cleared pathogenic SIV infections in Rhesus macaques (Hansen et al., 2013). Deleting the pp71 homolog within the RhCMV genome (Rh110) generated a vaccine vector that replicated well *in vitro* but poorly *in vivo*. However, the immunogenicity of the vaccine and its protection against SIV infection remained similar to wild type (Hansen et al., 2019; Marshall et al., 2019). Thus, generating an attenuated virus by removing a viral inhibitor of intrinsic immunity seems to have created a safer vaccine vector.

## Summary

Considerable effort on the part of many laboratories has established that pp71 is a multifunctional protein that regulates assorted cellular and viral processes. The stimulation of viral IE transcription via the inactivation of intrinsic immune defenses through Daxx degradation is the critical function of pp71 during productive replication, but is absent during latency because of the cytoplasmic sequestration of tegument-delivered pp71. Other functions of pp71 in regulating cell cycle progression and evading innate and adaptive immune responses appear to be largely dispensable for productive viral replication in fibroblasts, likely due to functional redundancy provided by other viral proteins. These functions of pp71 may play more important roles in other cell types or infection contexts. Mechanistic details of many of pp71 functions remain elusive. While it is clear that pp71 degrades at least a subset of its targets in a proteasome-dependent, ubiquitin-independent manner, the details of this process have yet to be illuminated. The subcellular localization of pp71 is clearly an important control point in regulating its function that appears to be dictated by both phosphorylation and the ill-defined process of virion uncoating, but specifics are lacking. The continued exploration of the functions and regulation of pp71 promises to reveal new insights into the sensing of incoming viral genomes, viral genome chromatinization and epigenetic modification, and how the modulation of cell cycle progression and each arm of the immune system contributes to the significant pathogenesis associated with HCMV infections.

## Author Contributions

All authors listed have made a substantial, direct and intellectual contribution to the work, and approved it for publication.

### Conflict of Interest

The authors declare that the research was conducted in the absence of any commercial or financial relationships that could be construed as a potential conflict of interest.
